# Syphilis: Its Relation to Scrofula

**Published:** 1882-09

**Authors:** John B. Roberts


SYPHILIS—ITS RELATION TO SCROFULA.—DR. JOHN B. ROBERTS (*Medical Times*, March 25, 1882,) presents the following views:

I. Syphilis and scrofula undoubtedly show a tendency to affect similar structures, as is proved by the many diseases formerly classified as scrofulous now known and admitted to be inherited syphilis. In fact, those who hold that the two affections are distinct are obliged sometimes to admit that it is sometimes impossible to determine from symptoms, or even from the antecedent history obtainable, which disease is responsible for the lesions exhibited.

II. Scrofula has been, and is, most common at times of life, in historic periods, and in countries in which syphilis is known to have been and to be prevalent and virulent. Again, scrofulous diseases are less prominent and more benign at the present day than formerly, when acquired syphilis was more destructive, because less judiciously treated.

III. It is so well known that syphilitic parents have, as a rule, syphilitic children that an exception gives rise to scientific comment. Scrofula is usually an inherited condition, and could readily, therefore, be due to such a widespread and prolific parentage as syphilis.

IV. Scrofulous affections are found in the highest stations of life—a fact which would be well nigh inexplicable if scrofula were the result of exposure, insufficient food, filth and bad air. If it be a grandchild of syphilis, however, its presence in every household can be as general as the immorality that gives birth to ts ancestor.

V. Cases occur similar to that reported by Lugol, who refers to a family of two healthy children, the father of whom subsequently contracted syphilis and inoculated his wife. His third child was born, who, after exhibiting many scrofulous symptoms, died at eighteen years, though the elder brother and sister presented no such conditions of ill health.

VI. Acquired syphilis has been accused of awakening scrofulous manifestations in those predisposed to scrofula in whom no symptoms have occurred for years. This suggests a relationship between the affections which is very intelligible if scrofula be viewed as a remotely inherited syphilis.

VII. Scrofula is the condition furnishing deposits of tubercle, which are the result of infection arising especially from cheesy accumulations. Syphilis is an incessant instigator of inflammations and of gummy tumors which frequently undergo caseous degeneration. Hence pathology points at syphilis as an agent well calculated to give birth to the scrofulous diathesis.

VIII. The most effectual remedies in the treatment of syphilis, whether acquired or inherited, are mercurials and preparations containing iodine. These so-called specifics in the management of syphilis are agents of greatest benefit in alleviating scrofulous manifestations.

The arguments of those denying the syphilitic parentage of scrofula may be summarized as follows:

I. Scrofula is not inoculable, as are the primary, the secondary, and sometimes the inherited lesions of syphilis.

II. The successive steps between primary syphilis in the progenitor and scrofulous diseases in the posterity have not been traced by any scientific investigators, who quote only isolated cases of apparent relationship.

III. Scrofulous affections are found in patients who have presented no such manifestations in early life (when inherited syphilis is especially noticeable), who have had neither scrofulous nor syphilitic parentage, and who had never acquired primary syphilis. Moreover, phthisis, which is a lesion of scrofula, is found very frequently in animals not known to have syphilis.

IV. Scrofula is not considered a factor in the causation of syphilis, as might be expected if there was a mutual relationship between them.

V. Scrofula is believed by some to have existed in Europe before the advent of syphilis, and according to certain writers is prevalent in countries where syphilis is rare.

VI. Syphilis yields more quickly and readily than scrofula, though the same or similar remedies may be available in both diseases.

Such are the arguments of the advocates and of the opponents of the syphilitic origin of scrofula. The affirmative side seems to me to present more points than are generalizations, and, at the same time, to offer greater difficulties for its opponent to meet. The negative arguments are weakened by the facts—1, that most animal poisons become weakened when passed through several subjects, and that is evidence pointing to the possibility of scrofulous disease being transferred from one person to another; 2, that the links of the chain must be imperfect when the period of hereditation and differentiation requires probably several generations; 3, that it is admitted even by the affirmative side that scrofula may arise from ordinary inflammations, and hence may occur in animals as well as in a non-syphilized man; 4, that many causes in nature cannot assume the rôle of effects; 5, that statistical evidence is proverbially subject to errors; and, 6, that the relative time of therapeutic influences depends more on the tissue-changes produced than on the etiological agency.

A survey of the field has led me to the conclusion that vast numbers of cases of so-called scrofulous disease are the direct result of inherited syphilis, but that, until pathology ascribes the tuberculous diathesis to a *special* and not a mere inflammatory infection, it is illogical to deny that acquired, and therefore also hereditary, scrofula may, and at times does, arise from absolutely non syphilitic precedents.—*Detroit Lancet.*

